# 超高效液相色谱-二极管阵列检测器串联电雾式检测器法测定化妆品中6种美白功效成分

**DOI:** 10.3724/SP.J.1123.2025.09016

**Published:** 2026-05-08

**Authors:** Xiaofang LI, Ye WANG, Fan FAN, Wei ZHOU, Bin CHEN, Huajin SHI, Guoqiang CAI, Ying LIU, Yibo HE

**Affiliations:** 1.纳爱斯浙江科技有限公司，浙江 杭州 310056; 1. NICE Zhejiang Technology Co. Ltd. ，Hangzhou 310056，China; 2.南京野生植物综合利用研究所，江苏 南京 211100; 2. Nanjing Institute for Comprehensive Utilization of Wild Plants，Nanjing 211100，China; 3.赛默飞世尔科技（中国）有限公司，上海 201206; 3. Thermo Fisher Scientific （China） Co. Ltd. ，Shanghai 201206，China; 4.纳爱斯集团有限公司，浙江 丽水 323000; 4. NICE Group Co. ，Ltd. ，Lishui 323000，China

**Keywords:** 超高效液相色谱, 二极管阵列检测器, 电雾式检测器, 美白成分, 化妆品, ultra performance liquid chromatography（UPLC）, photodiode array detector（PDA）, corona charged aerosol detector （CAD）, whitening ingredients, cosmetics

## Abstract

建立了一种超高效液相色谱-二极管阵列检测器串联电雾式检测器（UPLC-PDA-CAD）测定化妆品中乙酰壳糖胺、氨甲环酸、烟酰胺、苯乙基间苯二酚、光甘草定及抗坏血酸四异棕榈酸酯6种美白成分的分析方法。样品用二氯甲烷和水涡旋提取，收集二氯甲烷层和水层；水层经二氯甲烷洗涤后，合并二氯甲烷相，氮吹浓缩至<1 mL并以异丙醇定容。水层和异丙醇定容液分别过滤，注入液相色谱仪，通过Waters HSS T3柱（150 mm×2.1 mm，1.7 μm）分离。流动相为异丙醇-乙腈-20 mmol/L乙酸铵缓冲液（甲酸调节pH至4.0），采用梯度洗脱方式，以二极管阵列检测器串联电雾式检测器进行检测，外标法定量。分别对样品前处理方法和色谱条件进行了优化。在优化的实验条件下，6种美白功效成分在一定范围内线性关系良好，相关系数（*r*）均>0.999，在乳、霜、油3种化妆品基质中的加标回收率为92.8%~110.1%，相对标准偏差（RSD，*n*=6）为0.12%~5.54%。对市售的7款化妆品进行检测，检出的美白成分与产品包装标示成分一致，但各成分含量差异较大。烟酰胺的使用频率和测得含量均较高，5款产品的测试结果为0.19%~2.29%。该方法操作简便，稳定可靠，重复性好，适用于化妆品中这几种极性差异显著的美白成分的检测。

## Abstract (en)

Whitening is a core function of cosmetics， and ingredients including acetyl glucosamine， tranexamic acid， nicotinamide， phenethyl resorcinol， glabridin， and ascorbyl tetraisopalmitate are widely used due to their clearly established whitening mechanisms. However， existing analytical methods face notable limitations： photodiode array detectors （PDA） cannot reliably detect weakly or non-ultraviolet （UV）-absorbing components； evaporative light scattering detectors （ELSD） suffer from inadequate sensitivity for low-concentration analytes； mass spectrometry （MS） is costly； and single-column cannot achieve the separation of ingredients with significantly different polarities. To address these challenges， this study established an analytical method for the determination of the six whitening ingredients in cosmetics using ultra performance liquid chromatography coupled with a photodiode array detector and a corona charged aerosol detector （UPLC-PDA-CAD）. For sample pretreatment： 0.2 g of sample was weighed， and 10 mL dichloromethane and 10 mL deionized water were added for vortex extraction of whitening ingredients. The mixture was then centrifuged for phase separation. The aqueous layer and dichloromethane layer were collected separately. The aqueous layer was washed twice with dichloromethane； all dichloromethane fractions were combined， concentrated to less than 1 mL under a nitrogen stream， and finally made up to volume with isopropanol. Both the aqueous layer and the reconstituted isopropanol solution were filtered through 0.22 μm hydrophilic polytetrafluoroethylene （PTFE） membranes before UPLC injection. For chromatographic separation， a Waters HSS T3 column （150 mm×2.1 mm， 1.7 μm） was selected. The column temperature was set at 40 ℃， the injection volume was 1 μL， and the flow rate was 0.3 mL/min. The mobile phase consisted of isopropanol， acetonitrile， and 20 mmol/L ammonium acetate solution （pH adjusted to 4.5 using formic acid）， using a gradient elution program. For detection， a tandem PDA-CAD system was used： PDA （scan range 200–400 nm） was employed for components with UV absorption， while CAD （nebulization temperature 35 °C， acquisition frequency 5 Hz） was used for components with weak UV absorption. Quantification was performed using the external standard method.Method validation results showed good linear relationships for all six whitening ingredients within their respective concentration ranges. The correlation coefficients （*r*） were all greater than 0.999. The limits of detection （LODs， *S/N*=3） were 5.0–50.0 µg/g， and the limits of quantification （LOQs， *S/N*=10） were 12.0–120.0 µg/g. Spiked recovery tests were conducted on negative cosmetic matrices （emulsion， cream， oil） at low， medium， and high levels. The recoveries ranged from 92.8% to 110.1%， and the relative standard deviations （RSDs， *n*=6） were 0.12%–5.45%， indicating excellent precision and accuracy. This method was applied to seven commercially cosmetics. The results revealed that all target whitening ingredients declared on the product labels were detected， with significant differences in their content. Nicotinamide was the most frequently detected compound， found in five products， and also exhibited the highest concentrations， ranging from 0.19% to 2.29%. Phenethyl resorcinol was detected in three products， with contents ranging from 0.02% to 0.52%. Ascorbyl tetraisopalmitate was detected in two products， at 0.09% and 3.08%， respectively. Acetyl glucosamine， tranexamic acid， and glabridin were each detected in only one product. In conclusion， this established UPLC-PDA-CAD method is simple， efficient， sensitive and accurate. It effectively overcomes the technical challenge of detecting whitening ingredients with widely varying polarities， offering reliable technical support for cosmetic quality control， regulatory supervision， and evaluation of product whitening efficacy and potential sensitization risks.

美白作为化妆品的核心功效之一，主要通过抑制酪氨酸酶活性、阻断黑色素转运、抗氧化等机制改善肤色^［1-6］^，现已成为消费者首要诉求，市场规模持续扩大。烟酰胺、苯乙基间苯二酚、光甘草定等成分因美白功效机制明确，而被广泛应用于乳液、膏霜、油类等各类化妆品中^［6，7］^。例如，烟酰胺抑制黑色素小体转运，氨甲环酸间接抑制黑素细胞活化与黑素释放，乙酰壳糖胺可促进角质代谢，光甘草定和间苯二酚类衍生物则通过抑制酪氨酸酶活性减少黑色素生成^［8］^。这些成分的合理添加是产品功效的保障，因此建立高效准确的检测方法对产品质量控制至关重要。

目前，化妆品中美白成分的检测以色谱及色谱-质谱联用技术为核心^［1，4，9］^，其在中国市场遵循的主要法规依据有2015版《化妆品安全技术规范》^［10］^和相关国家标准（如GB/T 35954-2018和GB/T 30926-2014等）。具体检测技术中，高效液相色谱（HPLC）及超高效液相色谱（UPLC）方法是主流方法，其核心在于通过优化色谱条件与检测器，以满足不同极性目标成分的分离和检测需求。例如针对具有紫外吸收的烟酰胺、苯乙基间苯二酚、光甘草定等，二极管阵列检测器（PDA）因可选择性提取特征波长（200~400 nm）信号而被广泛使用^［11-15］^；而对于乙酰壳糖胺这类仅在紫外末端有弱吸收的强极性成分，目前化妆品领域暂未查询到相关文献，仅在药物分析领域有通过通用型蒸发光散射检测器（ELSD）检测的案例，但该方法检出限较高（>100 mg/L）^［16］^。色谱柱选择上，传统C_18_柱多用于弱极性成分分离^［17，18］^，而针对强极性成分，通常采用亲水作用色谱柱增强保留^［19］^，或者通过衍生化反应提高待测组分在C_18_柱上的保留能力^［20，21］^。此外，液相色谱-质谱联用（LC-MS）方法凭借高特异性与高灵敏度，在复杂基质的痕量成分检测中具有优势，可通过多反应监测（MRM）模式或平行反应监测（PRM）模式准确定量，尤其适用于低浓度美白功效成分的验证^［22-25］^。

然而，现有方法仍存在明显局限性：一是检测器适配性不足，PDA无法覆盖无紫外或弱紫外吸收成分，ELSD因灵敏度较低难以满足低浓度样本检测需求，质谱仪器成本较高且对弱极性成分的离子化效率较低，普及性受限；二是色谱分离面临挑战，单一色谱方法难以兼顾几种极性差异显著的成分（强极性成分如乙酰壳糖胺、氨甲环酸；弱极性成分如抗坏血酸四异棕榈酸酯），传统C_18_色谱柱对强极性成分保留较弱，易出现峰形展宽或共流出现象，而亲水作用色谱柱则难以兼顾弱极性成分的保留。

针对上述问题，本研究拟选取6种极性差异较大的美白成分作为目标物，包括乙酰壳糖胺、氨甲环酸、烟酰胺、苯乙基间苯二酚、光甘草定和抗坏血酸四异棕榈酸酯。采用超高效液相色谱-二极管阵列检测器串联电雾式检测器建立6种美白成分的检测方法，以期为化妆品中多组分美白成分的检测提供新思路，为化妆品的产品质量控制和有效监管提供技术支持。

## 1 实验部分

### 1.1 仪器、试剂与材料

ACQUITY H-Class超高效液相色谱仪（带二极管阵列检测器）（美国Waters公司）；Corona Veo RS电雾式检测器（美国Thermo Fisher Scientific公司）；HN-64全自动氮吹仪（上海新仪微波化学科技有限公司）；MS105电子天平（瑞士梅特勒托利多公司）；海道夫Multi Reax涡旋混匀器（德国Heidolph公司）；KQ5200DE超声波清洗器（昆山超声仪器公司）；H1750R高速冷冻离心机（湖南湘仪实验室仪器开发有限公司）；PURELAB Flex纯水仪（英国ELGA LabWater公司）。

乙腈（ACN，色谱纯，德国Merck公司）；异丙醇（IPA，质谱级，德国Merck公司）；乙酸铵和甲酸（色谱纯，美国Fisher公司）；二氯甲烷（DCM，分析纯，科隆化学品）；乙酰壳糖胺（≥99.5%）、氨甲环酸（≥98%）购自Sigma-Aldrich；烟酰胺（≥99.8%）、苯乙基间苯二酚（≥98%）、光甘草定（≥99%）、抗坏血酸四异棕榈酸酯（≥95%）均购自阿拉丁生化科技；亲水聚四氟乙烯（PTFE）滤膜（0.22 μm，上海安谱科学仪器有限公司）。实验用水为超纯水（H_2_O，电阻率为18.2 MΩ·cm）；化妆品样品均购自本地市场，包括乳、油、霜和膏4种基质。

### 1.2 对照品溶液配制

亲水性标准品配制：分别精确称取100 mg乙酰壳糖胺、氨甲环酸和烟酰胺标准品于3个50 mL容量瓶中，加去离子水溶解，并定容至刻度，得到质量浓度为2 mg/mL的单标储备液。采用铝箔避光保存于4 ℃冰箱中，有效期180天。将以上单标储备液用去离子水逐级稀释，配制成系列混合标准工作溶液，使乙酰壳糖胺和氨甲环酸的质量浓度为10、50、100、200、400 μg/mL，烟酰胺的质量浓度为10、50、125、250、500 μg/mL。此为混合标准工作溶液A，临用前配制。

亲脂性标准品配制：分别精确称取100 mg苯乙基间苯二酚、光甘草定和抗坏血酸四异棕榈酸酯标准品于3个50 mL容量瓶中，加入异丙醇溶解，并定容至刻度，得到质量浓度为2 mg/mL的单标储备液。采用铝箔避光保存于4 ℃冰箱中，有效期180天。将上述单标储备液用异丙醇逐级稀释，配制成系列混合标准工作溶液，使苯乙基间苯二酚和光甘草定的质量浓度为5、20、50、100、200 μg/mL，抗坏血酸四异棕榈酸酯的质量浓度为10、100、250、500、1 000 μg/mL。此为混合标准工作溶液B，临用前配制。

### 1.3 样品前处理

精确称取待测化妆品样品0.2 g（精确至0.000 1 g）至50 mL离心管中，乳液和油类样品先经氮吹处理，膏霜类样品无需氮吹处理。准确加入10 mL二氯甲烷，涡旋5 min进行充分提取，再准确加入10 mL去离子水，涡旋10 min后，8 000 r/min离心5 min。收集二氯甲烷层和水层，水层用10 mL二氯甲烷分两次洗涤，合并所有二氯甲烷液，氮吹浓缩至<1 mL，加异丙醇复溶并定容至10 mL。移取水层提取溶液和异丙醇复溶液分别过0.22 μm亲水PTFE滤膜，作为供试品溶液A和供试品溶液B，待进样分析。

不同化妆品样品中6种美白成分含量差异较大，上述取样量按照微量进行操作，针对高于标准曲线范围的样品可以减小取样量，或者将待测液进一步稀释；针对低于标准曲线范围的样品可以增大取样量，或者减少萃取溶剂用量。

### 1.4 仪器条件

采用Waters HSS T3色谱柱（150 mm×2.1 mm，1.7 μm），柱温为40 ℃，进样量为1 μL，流速为0.3 mL/min，检测器为PDA串联CAD。PDA扫描范围200~400 nm，其中烟酰胺的检测波长为260 nm，苯乙基间苯二酚和光甘草定的检测波长为280 nm，抗坏血酸四异棕榈酸酯的检测波长为220 nm，采集频率为5 Hz。CAD检测器检测乙酰壳糖胺和氨甲环酸，雾化温度为35 ℃，采集频率为5 Hz；流动相A为异丙醇，B为乙腈，C为20 mmol/L乙酸铵缓冲溶液（甲酸调节pH至4.0），梯度洗脱，洗脱程序详见表1。

**表1:** 梯度洗脱条件

Step No.	Time/min	*φ*（Isopropanol）/%	*φ*（Acetonitrile）/%	*φ*（Buffer）/%
1	0	0	2	98
2	2.5	0	2	98
3	12	0	45	55
4	16	60	40	0
5	18	85	15	0
6	25	85	15	0
7	26	0	2	98

Buffer： 20 mmol/L ammonium acetate buffer solution （adjusted to pH 4.0 using formic acid）.

## 2 结果与讨论

### 2.1 仪器条件优化

#### 2.1.1 检测器的选择及优化

在200~400 nm波长范围内对6种化合物进行扫描，结果显示烟酰胺、苯乙基间苯二酚、光甘草定和抗坏血酸四异棕榈酸酯具有紫外吸收，可选择的最大吸收波长分别为260、280、280、220 nm，适合采用PDA进行检测；乙酰壳糖胺和氨甲环酸仅在紫外末端有弱吸收，需用通用型检测器。实验对比了ELSD与CAD对这两种成分的检测性能，表明采用CAD时的检出限（1 μg/mL）显著优于ELSD（50 μg/mL），更适合化妆品中低浓度成分的检测。因此，最终选择将PDA和CAD进行串联的仪器方法，PDA扫描波长200~400 nm，按260 nm（烟酰胺）、280 nm（苯乙基间苯二酚和光甘草定）、220 nm（抗坏血酸四异棕榈酸酯）提取检测信号，检测4种成分；CAD检测氨甲环酸和乙酰壳糖胺。

#### 2.1.2 色谱柱的选择

针对6种美白成分的极性差异，本实验系统比较了Waters BEH C_18_（150 mm×2.1 mm，1.7 μm）、Waters BEH Amide（150 mm×2.1 mm，1.7 μm）和Waters HSS T3 （150 mm×2.1 mm，1.7 μm）3种不同色谱柱的分离性能，典型CAD检测谱图见图1，PDA提取谱图见图2。从图1可以看出，乙酰壳糖胺在Amide柱上出现了分叉，而氨甲环酸则由于保留太强未出峰；两种极性成分在C_18_柱上不能实现基线分离；相对而言，HSS T3的分离效果略好于C_18_柱，前者对乙酰壳糖胺和氨甲环酸的保留能力均变强，实现基线分离。由图2可知，Amide柱对烟酰胺表现出较好的保留能力，但剩余3种弱极性目标物苯乙基间苯二酚、光甘草定和抗坏血酸四异棕榈酸酯在Amide柱上均无有效保留，出现了色谱峰完全重叠现象。另外，在同样的色谱条件下，HSS T3柱对烟酰胺的保留能力明显强于C_18_柱，而两者对苯乙基间苯二酚和光甘草定的保留能力接近，均能满足分离要求。但抗坏血酸四异棕榈酸酯在HSS T3和C_18_柱上均未能出峰，推测可能是该化合物极性较小、与固定相作用力较强导致的。综上，本实验选择Waters HSS T3色谱柱进行分离优化。

**图1:**
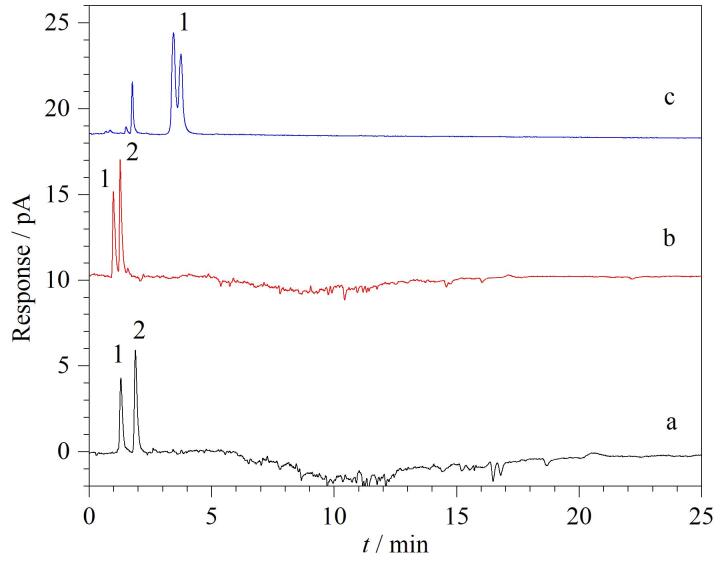
采用CAD检测器时乙酰壳糖胺和氨甲环酸在不同色谱柱上的色谱图

**图2:**
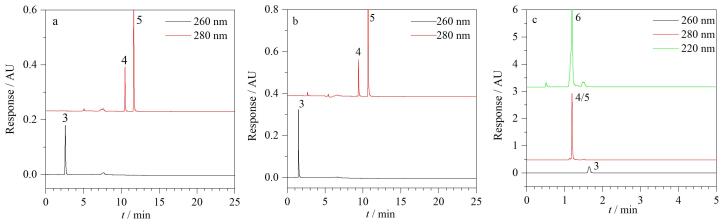
采用PDA检测器时4种美白成分在不同色谱柱上的色谱图

后续色谱条件的主要优化方向如下：调整流动相起始比例，使乙酰壳糖胺和氨甲环酸的保留时间尽可能延长，同时提高两者的分离度；进一步加强淋洗程序后半部分流动相的洗脱能力，使抗坏血酸四异棕榈酸酯顺利出峰并不受其他物质干扰。

#### 2.1.3 流动相的优化

利用液相色谱法分析美白功效成分，文献和相关标准多采用磷酸二氢钾-甲醇或磷酸二氢钾-乙腈作为流动相^［26-28］^，但由于CAD不可使用非挥发性盐^［29］^，故本实验选用可挥发的乙酸铵缓冲溶液作为水相缓冲盐，乙腈作为有机相。实验对比了初始流动相中20 mmol/L乙酸铵缓冲溶液（甲酸调节pH至4.5）体积占比为100%、98%和95%时，乙酰壳糖胺和氨甲环酸的分离情况，结果见图3。当初始为100%缓冲盐时，乙酰壳糖胺的色谱峰轻微分叉，推测可能与吡喃环的异构有关^［30，31］^；当初始比例为98%和95%时，乙酰壳糖胺的色谱峰形正常无分叉。进一步对比可见，初始缓冲盐比例为98%时，乙酰壳糖胺和氨甲环酸的分离效果更佳，实现基线分离。因此，选择缓冲盐初始比例为98%。

**图3:**
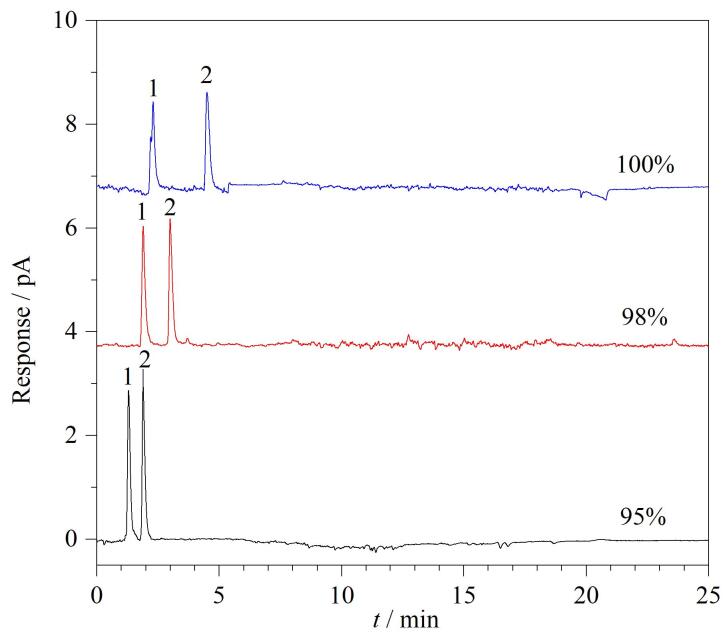
采用CAD检测器时乙酰壳糖胺与氨甲环酸在不同乙酸铵缓冲溶液体积分数下的色谱图

实验表明，乙酰壳糖胺、氨甲环酸和烟酰胺在HSS T3柱上的保留时间随缓冲盐pH值的变化而变化，如图4所示。在pH 3.0~6.0范围内，随着pH降低，乙酰壳糖胺和氨甲环酸保留值略有增强，烟酰胺则逐渐减弱。当缓冲盐pH用甲酸调整至4.0时，三者的保留与分离效果均较理想。弱极性的抗坏血酸四异棕榈酸酯仅用乙酸铵-乙腈体系无法洗脱，加入异丙醇后可有效改善其洗脱效果。最终确定流动相组成：A相异丙醇、B相乙腈、C相20 mmol/L乙酸铵缓冲溶液（甲酸调节pH至4.0），按1.4节中表1的梯度洗脱条件，6种美白成分的标准溶液谱图如图5所示。

**图4:**
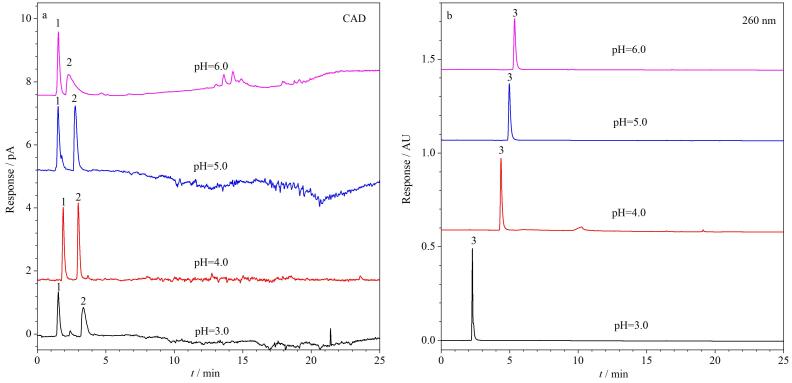
3种水溶性美白成分在不同pH值缓冲盐条件下的色谱图

**图5:**
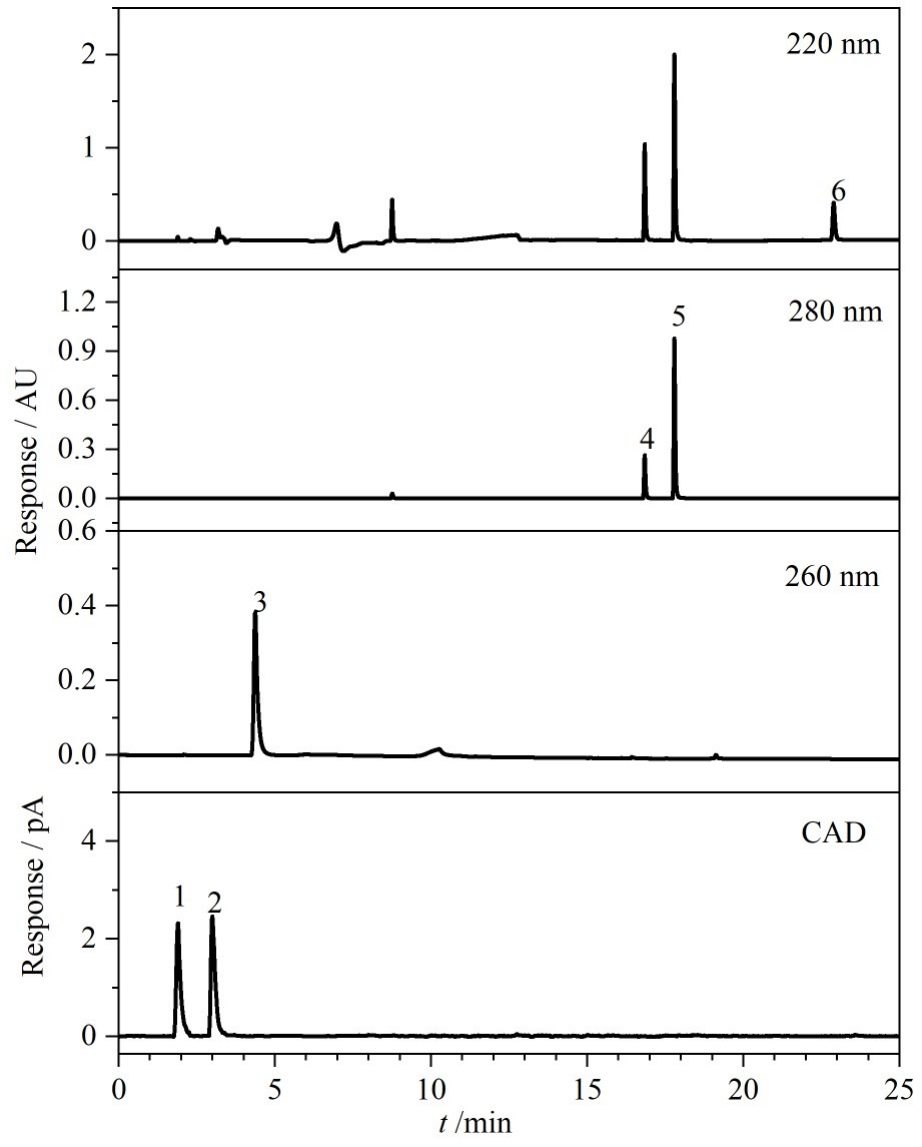
6种美白成分的色谱图

### 2.2 前处理条件优化

乙酰壳糖胺、氨甲环酸和烟酰胺为强极性化合物，易溶于水；而苯乙基间苯二酚、光甘草定和抗坏血酸四异棕榈酸酯为弱极性化合物，难溶于水，易溶于有机溶剂。实验对比了甲醇-水（1∶1，体积比）、乙醇（EtOH）、二氯甲烷-水（1∶1，体积比）3种萃取剂的提取效率（图6）。其中使用甲醇-水提取时，操作步骤为加入10 mL 甲醇-水涡旋振荡10 min，离心过滤得到待测液；使用乙醇提取时，直接加入10 mL无水乙醇溶液，涡旋振荡10 min，离心过滤得到待测液；使用二氯甲烷-水（1∶1，体积比）提取的操作步骤参照1.3节。

**图6:**
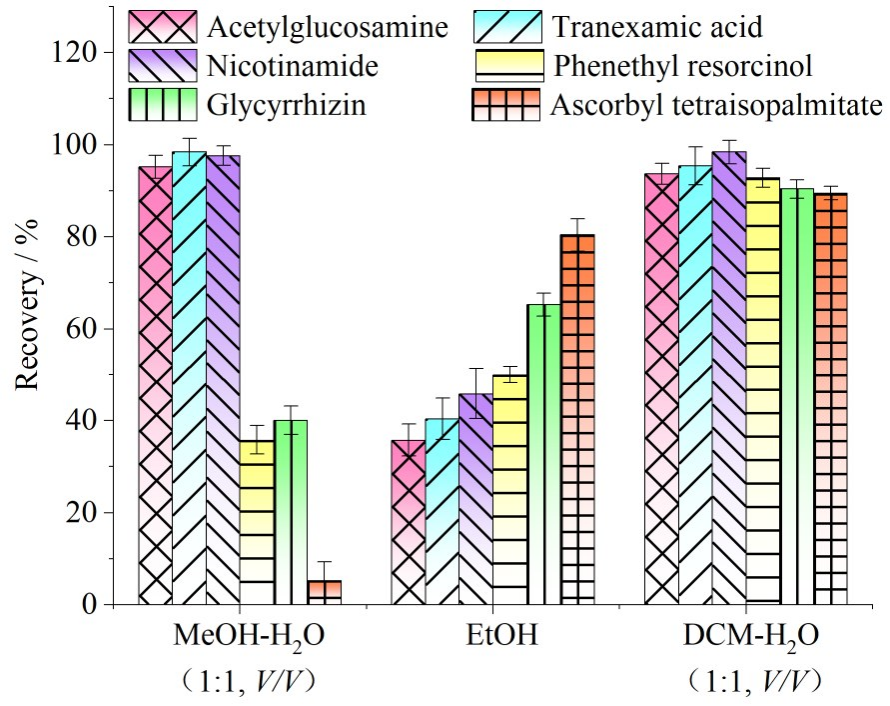
不同提取溶剂对6种美白成分回收率的影响（*n*=3）

结果表明，MeOH-H_2_O（1∶1，体积比）对苯乙基间苯二酚、光甘草定和抗坏血酸四异棕榈酸酯的回收率较差，尤其是抗坏血酸四异棕榈酸酯仅约5%，可能是因为三者在该体系中溶解性不足。EtOH提取的整体回收率不高，特别是几种强极性成分的回收率<50%，可能是溶剂效应导致色谱峰峰形变宽，回收率降低。而采用DCM-H_2_O（1∶1，体积比）体系提取时，目标物的回收率均>92.5%，可能得益于两相较大的极性差异——水相可有效萃取强极性成分，有机相可萃取弱极性成分。考虑到二氯甲烷可能损伤进样隔垫或色谱柱，有机相经氮吹浓缩后置换为高比例异丙醇；水层和异丙醇复溶液分别进样，既可避免高比例有机相导致强极性成分（如乙酰壳糖胺、氨甲环酸）出现溶剂效应，也可避免高比例水相导致弱极性成分（抗坏血酸四异棕榈酸酯）的溶解性和回收率不佳的问题。结合化妆品类型多样（乳、霜、油、膏等）且含有大量的油脂、表面活性剂和高分子物质，最终选择以下前处理方法：先用二氯甲烷分散样品并萃取出弱极性成分，再加入水超声涡旋萃取出强极性成分，最后对二氯甲烷层进行溶剂置换。

### 2.3 方法学验证

#### 2.3.1 线性范围和检出限

将1.2节配制的混合标准工作溶液A和B按照1.4节的色谱条件进行测定，每个浓度点测定3次，以目标化合物的质量浓度（*x*）为横坐标，其峰面积平均值（*y*）为纵坐标，绘制标准工作曲线并进行线性回归，得到各目标物的线性方程和相关系数，详细参数结果列于表2。结果表明，在一定范围内，6种美白成分的浓度与峰面积平均值呈现良好的线性关系，相关系数（*r*）均>0.999。为体现方法灵敏度，采用检出限（LOD）与定量限（LOQ）作为表征参数。在空白样品溶液中进行加标，并分别用超纯水和二氯甲烷进行逐级稀释，进样测得信噪比。以*S/N*=3计算检出限，以*S/N*=10计算定量限，当待测样品称样量为0.2 g，提取溶剂体积为10 mL，进样量为1 μL时，6种待测成分的检出限为5.0~50.0 µg/g，定量限为12.0~120.0 µg/g。

**表2:** 6种美白成分的线性方程、相关系数、线性范围、检出限和定量限（*n*=3）

Compound	Linear equation	*r*	Linear range/（μg/mL）	LOD/（μg/g）	LOQ/（μg/g）
Acetylglucosamine	*y*=152633*x*+4020156	0.9991	10-400	50.0	120.0
Tranexamic acid	*y*=180629*x*+4898788	0.9994	10-400	50.0	120.0
Nicotinamide	*y*=20152.26*x*-111428	0.9992	10-500	25.0	60.0
Phenethyl resorcinol	*y*=8237.66*x*-8067.87	0.9999	5-200	12.5	35.0
Glabridin	*y*=27133*x*-62060	0.9999	5-200	5.0	12.0
Ascorbyl tetraisopalmitate	*y*=6716.53*x*-169227	0.9999	10-1000	25.0	60.0

*y*： peak areas； *x*： mass concentration， μg/mL.

#### 2.3.2 回收率和精密度

以实验室自制的乳、霜、油3种化妆品阴性样本作为基质（称样量均为0.2 g）进行加标试验，6种美白成分的加标质量均为0.1、0.5和2.0 mg，以优化后1.3节的方法进行前处理，按照1.4节的色谱条件进行测定，并重复6次，计算各美白功效成分的加标回收率和相对标准偏差（RSD）。结果如表3所示，6种美白成分在3类不同化妆品中的加标回收率为92.8%~110.1%，RSD为0.10%~5.45%，表明该方法性能良好。

**表3:** 不同体系化妆品中6种美白成分的加标回收率和精密度（*n*=6）

Compound	Spiked/mg	Recoveries/%			RSDs/%		
		Emulsion	Cream	Oil	Emulsion	Cream	Oil
Acetylglucosamine	0.1	103.4	101.8	100.3	0.10	0.45	1.54
	0.5	97.2	102.0	101.5	0.21	0.43	5.45
	2.0	101.8	99.6	101.2	0.45	1.21	3.21
Tranexamic acid	0.1	101.1	100.4	108.7	0.54	1.54	1.58
	0.5	100.5	101.7	102.2	1.21	1.01	1.20
	2.0	99.5	99.0	98.3	0.45	0.89	0.98
Nicotinamide	0.1	99.9	97.2	95.6	0.48	1.21	1.21
	0.5	98.2	96.9	96.2	1.58	0.99	0.89
	2.0	97.3	95.7	96.7	2.15	0.12	0.54
Phenethyl resorcinol	0.1	98.1	105.4	103.8	4.58	1.01	0.57
	0.5	93.5	92.7	99.0	2.15	0.55	0.56
	2.0	110.1	103.9	102.4	1.20	0.22	0.85
Glabridin	0.1	98.9	104.3	101.1	3.51	0.99	0.78
	0.5	92.8	97.4	93.2	2.14	0.12	0.75
	2.0	94.4	102.3	100.9	0.21	0.35	0.89
Ascorbyl tetraisopalmitate	0.1	97.7	103.8	101.6	3.12	1.12	1.21
	0. 5	95.6	108.0	99.4	0.25	1.54	1.54
	2.0	100.7	109.2	104.5	0.21	1.56	3.69

#### 2.3.3 干扰试验

为了进一步确认化妆品样本中保湿剂和防腐剂是否干扰实验结果并产生假阳性，首先对待测实际样品的背标成分进行分析，筛选出配方中可能存在干扰的保湿剂、防腐剂和抗氧化剂等，包括甘油、乙基己基甘油、1，2-己二醇、戊二醇、苯氧乙醇、对羟基苯乙酮、苯甲酸钠、山梨酸钾、丁羟基甲苯和甘草酸二钾。实验对配制的包含上述10种成分和6种美白成分的混合溶液进行分析。如图7所示，其中甘油以及苯氧乙醇等7种成分与本文分析的6种美白成分基线分离，乙基己基甘油、1，2-己二醇和戊二醇未出峰，说明待测化妆品配方中的保湿剂、防腐剂和抗氧化剂等不会对测试造成干扰。

**图7:**
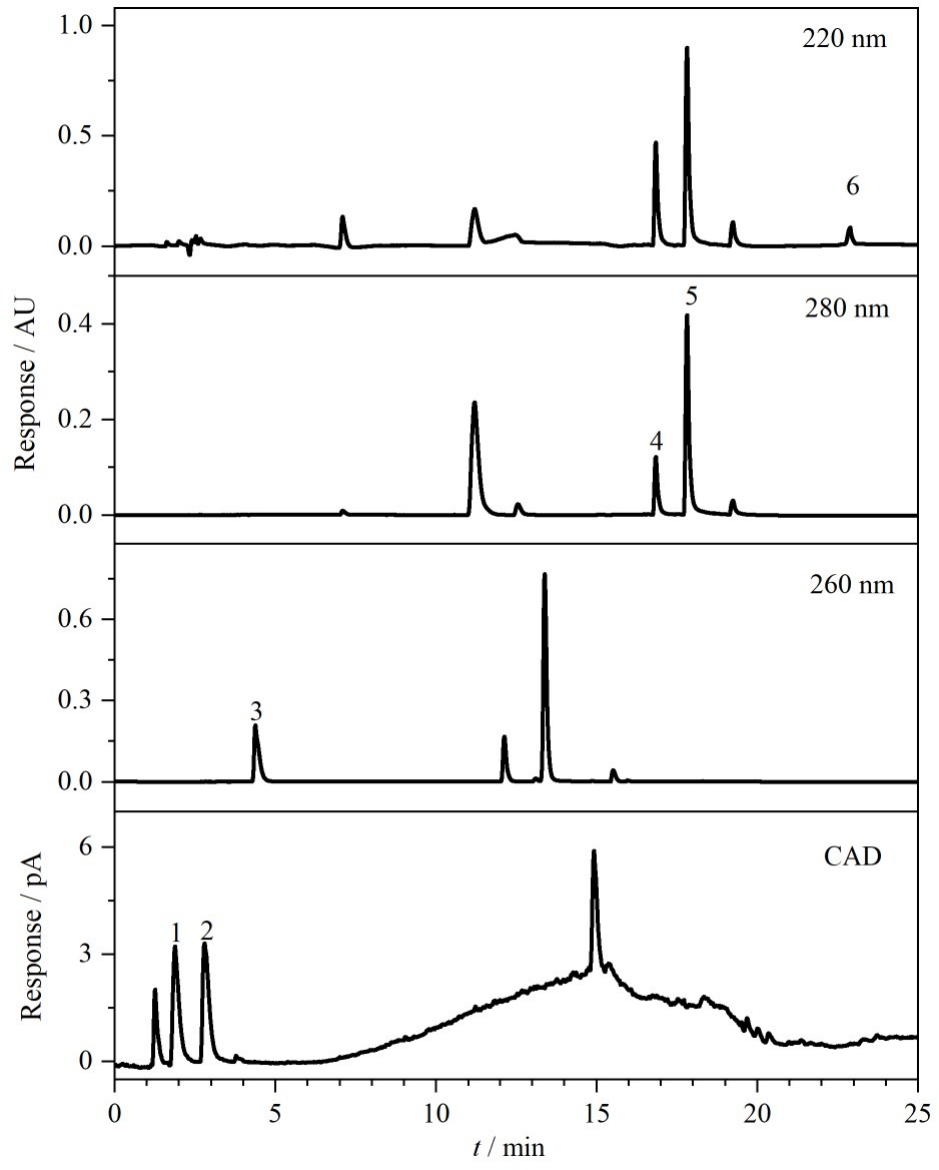
化妆品中常用保湿剂和防腐剂对6种美白成分的干扰测试色谱图

### 2.4 实际样本检测

对市售化妆品进行筛选，选取含本研究方法所涉及的1种及以上美白功效成分的产品作为实际测试试样，共获得7份。其中乳液类3份、油类1份、霜类2份、膏类1份。分别精密称取适量试样，依据1.3节样品前处理方法制备供试品溶液，采用1.4节的液相色谱法进行进样检测，再根据待测组分浓度判断是否稀释样品溶液，确保其处于标准曲线线性范围内，最后计算各样品中美白功效成分的含量，详细结果如表4所示。

**表4:** 7款化妆品样本中6种美白成分的检测结果（*n*=3）

Sample No.	Compound	Content/%
1	acetylglucosamine	1.54
	nicotinamide	2.29
2	tranexamic acid	1.61
3	nicotinamide	1.85
	phenethyl resorcinol	0.15
	glabridin	0.03
4	nicotinamide	0.25
	phenethyl resorcinol	0.52
5	nicotinamide	1.50
	ascorbyl tetraisopalmitate	0.09
6	nicotinamide	0.19
	phenethyl resorcinol	0.02
7	ascorbyl tetraisopalmitate	3.08

结果表明，本批实际样品检出的美白成分与产品包装中标注的美白成分均一致，但各成分含量差异显著。7款产品中，烟酰胺的使用频率和检测含量均较高，5款产品的测试结果为0.19%~2.29%；其次3款产品中检出苯乙基间苯二酚，含量为0.02%~0.52%；两款产品中检出抗坏血酸四异棕榈酸酯，分别为0.09%和3.08%；乙酰壳糖胺、氨甲环酸和光甘草定分别只在一款产品中检出。另外，样品2和样品7分别使用单一的美白成分，且含量相对较高；另外5款产品选择了两种及两种以上美白成分复配使用，其中样品1中两种美白成分含量相对均较高（1.54%和2.29%），而样品6中使用的两种美白成分含量均较低（0.19%和0.02%），其余3款基本为1个高浓度美白成分和1~2个低浓度美白成分搭配。

## 3 结论

本研究建立了一种超高效液相色谱-二极管阵列检测器串联电雾式检测器的检测方法，用于测定化妆品中乙酰壳糖胺等6种极性差异显著的美白功效成分。该方法操作简便，准确性高，可抗常规保湿剂、防腐剂等基质干扰；通过二氯甲烷和水分步提取，有效解决了强极性和弱极性成分萃取率偏低的问题；采用二极管阵列检测器和电雾式检测器串联，实现有/无紫外吸收成分的同步检测。实际样品检测结果表明，6种成分在乳、霜、油等化妆品中均能有效检出，可满足相关化妆品的质量控制需求，为当前化妆品美白功效成分的多组分检测提供了可靠的技术支撑。后续研究中可进一步拓展美白功效检测组分的范围，构建更全面的检测平台。
